# Effects of Delay, Question Type, and Socioemotional Support on Episodic Memory Retrieval by Children with Autism Spectrum Disorder

**DOI:** 10.1007/s10803-018-3815-3

**Published:** 2018-11-08

**Authors:** Telma Sousa Almeida, Michael E. Lamb, Emma J. Weisblatt

**Affiliations:** Department of Psychology, University of Cambridge, Free School Lane, Cambridge, CB2 3RQ UK

**Keywords:** Autism, Delay, Eyewitness testimony, Question types, Socioemotional support

## Abstract

Twenty-seven autistic children and 32 typically developing (TD) peers were questioned about an experienced event after a two-week delay and again after a two-month delay, using the Revised National Institute of Child Health and Human Development (NICHD) Investigative Interview Protocol. Recall prompts elicited more detailed and more accurate responses from children than recognition prompts. Autistic children recalled fewer correct narrative details than TD peers when questioned using open invitations, cued invitations, and directive questions. Nonetheless, they were as accurate as TD peers when responding to all types of prompts. The informativeness and accuracy of children’s reports remained unchanged over time. Social support was beneficial when children were interviewed for the first time but not after a longer delay.

Research in both field and experimental contexts has demonstrated that the methods and techniques used to retrieve memories of experienced and witnessed events—in other words, the type of questions posed—affect the structure of children’s responses and, particularly, the amount and accuracy of the information they provide (Lamb et al. 2015). As noted below, some researchers have explored how well autistic children and adults^1^ recall and describe events in response to a diverse range of questions soon after the events were witnessed or experienced, but it is unclear how detailed and accurate their recall is after longer delays between an event and subsequent reporting. One goal of the present research was thus to explore how well autistic children recalled an experienced event after delays of two weeks and two months. A further goal was to determine whether the amount and accuracy of information recalled varied depending on the supportiveness of the interviewer.

## Efficacy of Different Interviewer Prompts

Evidence from both field and experimental research has determined that broad open-ended prompts which tap recall memory processes, such as invitations (“Tell me everything that happened”) and cued invitations (e.g., “Earlier you mentioned […]. Tell me everything about that”) are associated with detailed, accurate, and uncontaminated descriptions of experienced events (see Lamb et al. 2015; Milne and Bull 1999 for reviews). However, extensive research investigating the specific memory profiles of individuals with Autism Spectrum Disorder (ASD) have shown that they often have difficulties when asked to freely recall information (Bennetto et al. 1996; Boucher and Warrington 1976; Bowler et al. 1997; Maras et al. 2012; McCrory et al. 2007). Such findings suggest that reliance on free recall prompts, as universally advocated by various protocols and professional guidelines for forensic interviewers (American Professional Society on the Abuse of Children 2012; Home Office 2011; Lamb et al. 2018), because they elicit elaborative narrative accounts, may be problematic for witnesses with ASD.

Some studies have shown that, soon after an event, children with ASD recall less information than TD peers in response to free-recall prompts, such as “Tell me everything that happened” (e.g., Bruck et al. 2007; Henry et al. 2017a, b; Mattison et al. 2015, 2016; McCrory et al. 2007). In addition, autistic children (e.g., Bruck et al. 2007) and adults (e.g., Maras and Bowler 2010, 2011, 2012) appear less accurate than TD peers when answering free recall questions shortly after witnessed or experienced events. In contrast, other researchers have reported that the free recall answers of autistic children can be as accurate as those of TD peers (Henry et al. 2017a, b; McCrory et al. 2007), particularly when they are supported by techniques such as the sketch-reinstatement-of-context (Henry et al. 2017b; Mattison et al. 2015, 2016) or Verbal Labels (Henry et al. 2017b). When supported by the physical context reinstatement technique (Maras and Bowler 2012), the free recall reports of autistic adults can similarly be as accurate as those of TD peers. Such findings suggest that free recall prompts can elicit accurate but less detailed information from autistic children and adults.

Directive questions, phrased as *wh*-questions (e.g., what, how, who, when, and where) also draw on recall processes and are often used in forensic interviews and in court (Andrews et al. 2015; Waterhouse et al. 2016; Yi et al. 2015). Directive questions involve cued recall, rather than free recall, sometimes refocusing children on previously disclosed details about the event but requesting specific categories of information. Cued recall often appears to be intact in individuals with ASD (Bennetto et al. 1996; Minshew et al. 1994), including when they are recalling experienced events (Bowler et al. 2008; Maras and Bowler 2010; Maras et al. 2012, 2013; McCrory et al. 2007; Millward et al. 2000).

Children’s recognition memory (i.e., the ability to decide whether a stimulus was or was not previously experienced or viewed) can be assessed using closed-ended yes/no, forced-choice, multiple-choice, and leading questions that request confirmation, denial, or selection among specific interviewer-generated options (i.e., option-posing questions). Studies assessing recognition memory in autistic individuals often show no significant differences in performance between ASD and TD individuals (Bowler et al. 2008; Lind and Bowler 2009a, b; Minshew and Goldstein 1993; Minshew et al. 1992).

Taken together, these findings suggest that directive (i.e., cued recall) and option-posing (i.e., recognition) questions might be appropriate when interviewing autistic children. However, it is now well-established that children provide less information in response to cued recall questions and closed questions that tap recognition memory than to free recall prompts, and that these questions are usually associated with more inconsistent and erroneous responses (Brown et al. 2013; Brown and Lamb 2015; Cederborg et al. 2000; Lamb and Fauchier 2001; Oates and Shrimpton 1991; Peterson et al. 1999; Waterman et al. 2000). In addition, comparable performance between children with and without ASD is not always evident in cued recall and recognition tests (e.g., Bruck et al. 2007; Millward et al. 2000).

To our knowledge, no studies have directly explored the effects of delay on responses to recall and recognition-based prompts and questions by autistic children. Previous studies investigating the effects of delay on TD children’s recall of non-stressful or staged events (as in the current study) have reported variability in performance over time in response to different types of prompts. Some studies have found that children’s accounts remain the same or even get better over time in response to free- and open-ended prompts (e.g., Bruck et al. 2002; Fivush and Hamond 1989; La Rooy et al. 2005 [Experiment 1 and 2]; Pipe et al. 2004) whereas others suggest that the amount and accuracy of the information retrieved decreases after longer delays in response to both free recall and recognition prompts (e.g., Baker-Ward et al. 1990; Hudson and Fivush 1991; La Rooy et al. 2005, 2009; [Experiment 3]; Pipe et al. 1999 [Experiments 1 and 2]; Salmon and Pipe 1997). These divergent findings are possibly related to methodological differences in how children’s reports were scored, because different studies have assessed different aspects of children’s memories (Peterson 2011).

In the course of a thorough interview, like a forensic interview, children are probed for information using a range of differently formulated recall and recognition-based prompts and questions. However, no research to date has specifically examined how children with ASD respond to different types of interviewer questions (i.e., unstructured, open-ended, free recall prompts versus specific cued recall, or close-ended and focused recognition prompts) after an extended delay, and how their responses to such types of questions change over time.

## The Role of Socioemotional Support

Over the past two decades, field and laboratory research has turned attention to how socioemotional factors affect children’s ability to provide evidence within legal proceedings (e.g., Goodman et al. 1991; Hershkowitz et al. 2009; Price et al. 2016). Developmental theories suggest that social support plays an important role in enhancing children’s cognitive performance (e.g., Fischer 1980) and the use of child-friendly interview techniques is currently advocated by researchers and professionals (e.g., Price et al. 2016; Saywitz et al. 2015; Vallano and Compo 2015). Establishing rapport and being supportive is important to gain children’s trust and cooperation in legal contexts (Aldridge and Wood 1998; Goodman and Bottoms 1993; Hershkowitz et al. 2006; Powell and Thomson 1994).

Some analogue research studies suggest that supportive and non-suggestive interviewing techniques, such as effective rapport-building using verbal and non-verbal socioemotional support can reduce TD children’s anxiety and discomfort, encouraging them to cooperate and provide elaborate and accurate accounts of past experiences (e.g., Bottoms et al. 2007; Goodman et al. 1991; Rush et al. 2014). Researchers have also demonstrated that support can decrease children’s suggestibility (e.g., Carter et al. 1996; Davis and Bottoms 2002; Goodman et al. 1991). However, in some laboratory analogue studies researchers have failed to find an effect of support on children’s responses to free recall and/or specific questions (e.g., Carter et al. 1996; Davis and Bottoms 2002; Imhoff 2000; Imhoff and Baker-Ward 1999). The discrepancies may be due to methodological differences in relation to the nature of the event (stressful versus neutral), the delay (e.g., 2 weeks, 4 weeks, 1 year), and the types of questions used to retrieve children’s memories.

In field studies, however, the results have been more consistent. Most research investigating suspected victims’ reports of child abuse suggests that interviewer supportiveness (e.g., non-suggestive positive reinforcement, addressing the child in a personal way, referring to the child’s emotions, facilitators) increases the amount and/or accuracy of the information provided (e.g., Hershkowitz 2009; Hershkowitz et al. 2006; Teoh and Lamb 2013; but see; Lewy et al. 2015).

Hershkowitz et al. (2013) explored whether supportive, non-suggestive techniques embodied in the recently developed Revised NICHD Protocol (the protocol used in the current study to retrieve children’s memories of the target event) would help alleged victims of intra-familiar abuse become more cooperative and less reluctant during forensic interviews (Ahern et al. 2014; Hershkowitz et al. 2013, 2014, 2017; Lamb et al. 2015). Children interviewed using the supportive Revised Protocol by Hershkowitz et al. (2013) were less reluctant and this was, in turn, associated with increases in the number of relevant details provided. Likewise, Ahern et al. (2014) found that supportive statements in the pre-substantive part of the same interviews studied by Hershkowitz et al. (2013) promoted children’s responsiveness and cooperation.

Overall, the available evidence seems to suggest that socially supportive interviewing conditions facilitate the disclosure of past experiences (especially abusive experiences) and increase the amount of information provided by children, without compromising accuracy. However, to our knowledge, no studies have examined the impact of interviewer-provided social support during investigative interviews on the memory performance of children with ASD.

## The Current Study

In the current study, we investigated how children with ASD responded to various types of interviewer prompts incorporated into the best-practice Revised NICHD Interview Protocol and how effectively different types of questions elicited new and accurate event-relevant information from children with ASD in two interviews. We were particularly interested in comparing the efficacy, after differing delays, of open-ended recall prompts, such as invitations and cued invitations, relative to more focused recall and recognition prompts (e.g., directive, option-posing and focused/ contaminating questions) in eliciting relevant information from children with ASD. Additionally, we examined the effects of interviewer supportiveness on the amount and accuracy of autistic children's accounts of personally experienced events, after differing delays. We explored how effectively supportive interviewer prompts elicited new and accurate event-relevant information, relative to prompts without social support, in the course of non-suggestive, child-oriented interviews.

Based on previous findings, it was predicted that children with ASD would report fewer narrative details than typically developing children in response to invitations and cued invitations (i.e., free recall prompts), but that there would be no difference between groups in responses to directive (i.e., cued recall prompts), option-posing, and focused/contaminating questions (i.e., recognition prompts). We expected that invitations and cued invitations would elicit more and more accurate narrative details from children in both groups than directive, option-posing, and focused/contaminating questions. Because no research has specifically examined the responses of children with ASD to different types of interviewer prompts after an extended delay, we made no predictions regarding their performance relative to TD children.

Also, informed by the research reviewed above, we predicted that supportive interviewer prompts would elicit more information than prompts not containing support, without compromising the accuracy of that information. Because some experimental studies involving interviews soon after the event have shown no effects of support on recall we expected that supportive prompts would be particularly effective in eliciting more detailed responses after a longer delay when children’s memory for the experienced event was less strong.

In light of the social interaction and communication deficits associated with ASD (APA 2013) two contrasting predictions could be made regarding whether children with ASD should benefit from, or even be receptive to, interviewer-provided social support. On the one hand, some children on the autism spectrum show little interest in and get anxious around other people, especially unfamiliar persons (Hobson 2002; Lord 1985, 1993). There are also evident of diminished social orienting, social seeking and liking, and social maintaining in ASD (see Chevallier et al. 2012 for a review), thus rendering social support during interviews unhelpful.

On the other hand, social interaction and cooperation by cognitively able children with autism can be enhanced using intervention strategies designed to foster social competencies (e.g., Bauminger 2002; Beaumont and Sofronoff 2008; Hagopian et al. 2009; Leaf et al. 2009). Thus, being supportive in uncertain and cognitively demanding situations, such as forensic interviews, could indeed help children with ASD feel more comfortable and less anxious, encouraging them to produce more complete and accurate accounts of past experiences.

## Method

### Sample

Fifty-nine 6–15 year-old children (mean = 9 years, 9 months) participated in the study (18 females and 41 males): 27 children with an ASD diagnosis who were able to verbally communicate and 32 typically developing children. The children with ASD were recruited from the Peterborough Integrated Children’s Health Services and the Cambridgeshire Community Services NHS Trust. Typically developing children were recruited from local schools in Peterborough and Cambridge. All children or their legal representatives provided informed consent and ethical approval for the study was obtained from the NHS Research Ethics Committee (NRES Committee East of England—Cambridge South).

All ASD participants (23 males and four females) had received (independently of the research study and 2 weeks before the research interview) a formal autism diagnosis by an appropriately qualified clinical professional. This diagnosis was obtained using the assessment criteria of the Autism Diagnostic Observation Schedule, Second Edition (ADOS-2; a cut-off point of 7 or 8), and the Autism Diagnostic Interview, Revised, which confirmed that the participants met DSM-V criteria for ASD (American Psychiatric Association 2013). After diagnosis, the children and their caregivers were informed about the study by their clinician at the Peterborough Integrated Children’s Health Services and given the relevant Participant Information Sheets and Consent forms. Children with ASD whose intellectual and linguistic abilities were within the normal range (verbal quotients of 85 or above; full-scale IQ of 90 or above—measured by the child clinician using the Wechsler Intelligence Scale for Children-Third Edition) and were interested in taking part in the study were then referred to us and contacted to set up the subsequent study sessions.

Thirty-two typically developing children (18 males, 14 females) were recruited through local mainstream schools in Peterborough and Cambridge. For each ASD participant, one or more typically developing child of the same chronological age was selected for the comparison group. They had no known psychiatric, developmental or neurological disorders, as indicated by parents/caregivers and the absence of symptomology to the date of the study. An independent *t test* confirmed that the groups did not differ significantly with respect to chronological age, *t*(57) = − 1.70, *p* = .095 (ASD: *M* = 10.63, SD   3.02, range  6–15 years; TD: *M* = 9.38, SD = 2.66, range  6–15 years).

## Materials and Procedure

This study was conducted in three phases. In phase one, children personally experienced an interactive live event and in phases two and three they were interviewed about this event using a best practice structured interview protocol, the first time after a short two-week delay and again after a longer 2-month delay.

### Phase One: Event-to-be-Recalled

The event-to-be-recalled was a set of activities included in the Autism Diagnostic Observation Schedule, Second Edition (ADOS-2). The ADOS-2 is a standardized instrument that assesses social interaction, communication, and imagination during a semi-structured interaction with an examiner (Lord et al. 2012). In this session, children engaged in a series of activities involving interactive stimulus materials. For children with ASD, a qualified psychiatrist conducted the ADOS-2 as part of the child’s diagnosis process, independently from the research study. This session occurred 2 weeks before children took part in the study. Typically developing children experienced the activities included in the ADOS-2 as part of the research study. A psychiatrist with prior ADOS-2 training conducted this session either at the Peterborough Integrated Children’s Health Services or at the University of Cambridge.

The activities that children engaged in during the event corresponded to Module 3 of the ADOS-2, with the exception of seven children with ASD who experienced Module 4 as per the clinician’s decision. The same tasks and materials comprised both modules 3 and 4. The examiners strictly followed the ADOS-2 manual and always provided the same instructions and displayed the same items, in the same way, and sequence, so the duration of the sessions (*M* = 44.63 min; range 40–53 min) depended only on the amount of time each child took to perform each task. Bivariate correlations revealed no significant relationships between the length of the event (ADOS-2 session) and the total number of unique narrative details recalled at the two-week interview by children in the ASD *r*(27) = − .03, *p* = .901, and TD *r*(32) = .16, *p* = .381 groups. The same results emerged for the two month interview with no significant relationships between the length of the event and the total number of unique narrative details recalled by children in the ASD *r*(59) = − .06, *p* = .773, and TD *r*(32) = .25, *p* = .161, groups. All event sessions were video-recorded, and the recordings were later used to determine the accuracy of the children’s accounts.

The event-to-be-recalled included a construction task, make-believe play, joint interactive play, a demonstration task, the description of a picture, telling of a story from a book, telling of a story depicted in cartoons, conversations about something that happened to the child in the past, questions about a variety of topics, a break, and the creation of a story using objects provided. Table 1 provides a detailed description, with examples, of the activities experienced during the event-to-be-recalled. Parents were asked not to discuss the event with their child because we were interested in what the children themselves remembered. On each subsequent session, parents confirmed not having talked with their child about what had happened during the event.

**Table 1:** Event to-be-recalled

Activities	Materials	Description
Construction task	Puzzle pieces and printed design to be duplicated	The examiner/researcher asked children to perform a construction task in which they assembled blocks to construct a design shown on a printed form
Make-believe play	Bag 1: 2 male action figures, 1 female action figure, 3 “prop” (one for each action figure), miniature hairbrush, 2 small tools, toy dinosaur Bag 2: small spoons and plates, several pieces of miniature food, small teapot/pitcher/measuring cup, miniature book, toy car, toy rocket, small ball, hologram disk, and two pieces of “junk” (small piece of cloth and small “jewellery”)	The examiner/researcher gave children some miniature play objects and instructed them to play with these materials
Joint interactive play	Materials from “Make-Believe” Play (Bags 1 and 2)	After a few moments the examiner joined in, making it clear that the play had become collaborative. The examiner/ researcher then told the children it was time to move on to the next activity and allowed them to help clean up the materials
Demonstration task	–	The examiner/ researcher asked children to demonstrate toothbrushing. The examiner drew with his/her finger a sink and faucet handles, a toothbrush, toothpaste, and a cup on the table in front of the participant and said: “Now I want you to show me and tell me how to brush your teeth. Start right at the beginning. You’ve just come into the bathroom to brush your teeth. What do you do now?”
Description of a picture	Option 1: Resort scene; Option 2: U.S. map scene	The examiner/researcher showed children a picture and asked them to describe it
Telling a story from a book	2 picture storybook for Modules 3 and 4	The examiner/ researcher asked children to recount a sequential story from a book of pictures “Let’s look at this book. It tells a story in pictures. See, it starts out with [first picture in the book]. Can you tell me the story as we go along? You go first. Then, I’ll take a turn.”
Cartoons	Series A: fisherman/pelican series Series B: monkey/coconut series	The examiner/ researcher showed the children a set of cards presenting a brief story in cartoon form, one frame per card, without any dialogue or narrative text. The examiner placed the cards one by one on the table and offered a brief statement describing the relevant setting. Children were then instructed to push their chair back from the table, stand up, and tell the story
Conversation and reporting	–	During the session, children were also encouraged to describe an event (e.g., a non-routine episode that had actually occurred (as opposed to an account of a film or story) such as a birthday party, a family celebration, a holiday, etc.)
Questions	–	The examiner/researcher asked children to answer a set of questions regarding a variety of topics. All children were asked the exact same questions about emotions (e.g., “what do you like doing that makes you feel happy and cheerful?”; “What about things that you’re afraid of?”), social difficulties and annoyances (e.g., “Are there things that other people do that irritate or annoy you?”; “Have you even been teased or bullied? Why, do you think?”), friends, relationships, and marriage (e.g., “Do you have some friends?”, “How is a friend different from someone whom you just go to school with?”), and loneliness (e.g., “Do you ever feel lonely?”). The seven children with ASD who experienced module 4 of the event were asked the same questions, as well as questions about responsibility (e.g., “Who takes care of your money”) and future plans and hopes
Break	Mini game (e.g., shape puzzle), drawing paper, set of eight markers, pin art, spin pen (spin top with pen base), small radio, current newspaper and magazine	The examiner/ researcher gave children a break during which he gave them some objects and materials and encouraged them to play freely. The break could occur at any time during the session
Creating a story	Six small objects with a definite purpose (e.g., umbrella, car), six small objects with no clear purpose (e.g., piece of string, wooden block)	The examiner/researcher asked children to create a story using a set of objects. A bag containing 12 small objects was provided. The examiner picked five things from the bag and used them to make up a story. After finishing his/her story, the examiner/researcher instructed the children to pick five different things from the bag and make up their own story

Event description and examples

### Phases Two and Three: Interviews

Children were interviewed about the personally experienced event twice, the first time after a short two-week delay and again after a longer two-month delay. Both interviews were conducted according to the best practice Revised NICHD Protocol developed by Lamb et al. (see Lamb et al. 2018 for a full discussion of the Protocol), by one of three interviewers (the first author, a licenced forensic psychologist with experience interviewing vulnerable witnesses using the NICHD Protocol; a graduate and a post-graduate psychology researcher, both with previous training in the use of the NICHD Protocol and experience of interviewing vulnerable interviewees). The two interviews were conducted by the same person, except in four cases where practical constraints prevented the same interviewer from conducting both interviews. One-way Analyses of Variance (ANOVA) showed no significant effect of Interviewer on the total number of narrative details reported by children in the two-week interview, *F*(2, 56) = 1.63, *p* = .204, or in the two-month interview, *F*(2, 56) = 0.89, *p* = .417.

This was the first study to use the NICHD Protocol (standard or revised versions) to interview children with ASD about events they had personally experienced. The NICHD Protocol is a structured, non-suggestive, and child-directed interview protocol and its recent revision incorporates advice for interviewers on how to build better rapport and provide children with more support throughout the interview. The NICHD Protocol has been systematically evaluated in the field, is currently used by forensic interviewers in several countries worldwide and is recommended to forensic investigators in the United Kingdom (Home Office 2011). The positive impact of using this Protocol to interview children in several countries has been examined recently (La Rooy et al. 2015) and it has been proven effective with different populations, including children as young as 3 years old (Hershkowitz et al. 2008, 2012; Lamb et al. 2003), and children with intellectual disabilities (e.g., Brown and Lamb 2015; Brown et al. 2012, 2015, 2017).

All interviews (at both time points) comprised the same phases in the same order, as follows: (1) greet; (2) rapport (3) ground rules, truth and lie exercise; (4) substantive recall part of the interview (i.e., interviewers’ statements or questions and children’s responses that pertained to the investigated event); and (5) closure. Children were assured that there were no wrong or right answers and that there were no time limits. While children were explaining what they could remember, the interviewer exhibited active listening and did not interrupt the child. Literal and concrete thinking is common in individuals with ASD, so interviewers framed each question/statement (for both groups of children) as directly, briefly, and clearly as possible to avoid providing too much information at once. Children were provided long wait/processing times after each question/statement to give them time to reflect on the questions and answers. This study focused on the information elicited during the *substantive portion of the interviews*, and so only this portion of the interview is described in detail below. We briefly describe the greet, truth and lie, rapport and closure phases, and the full interview protocol is available in Lamb et al. (2018).

Interviewers began by introducing themselves and establishing rapport and proceeded to clarify the children’s task (the need to describe experienced events truthfully and in detail) and explain the ground rules for the interview (i.e., that they could and should say “I don’t remember,” “I don’t know,” “I don’t understand,” or correct the interviewers when appropriate). In the rapport-building phase, children were prompted to provide information about personally meaningful topics using open-ended invitations (e.g., “Tell me about things you like to do”) and were encouraged to elaborate on their responses. They were then asked to describe in detail a recent event they had experienced (e.g., holiday, birthday party, first day at school, etc.) to practice retrieval of episodic memories and to further develop rapport. Here the interviewer introduced other types of questions that could be used when seeking information about the to-be-recalled event.

The *substantive* part of the interviews followed the structure outlined in the Revised NICHD Protocol. The interviewers used a series of open-ended recall prompts (e.g., invitations, follow-up invitations, cued invitations, *wh-*questions) to encourage children to provide as much information as they could remember about the event and children’s responses were used as cues for further recall. More focused prompts, such as yes/no questions, were avoided but used if needed to clarify unclear information and these were followed by open prompts (e.g., “Tell me more about that”). Once the child had finished speaking and was waiting for the next instruction, they were once again asked: “Is there anything else you remember?”. This prompt was repeatedly asked until the child could not offer further information.

In our study we included an additional questioning phase that is not part of the Revised NICHD Protocol. This questioning phase was implemented after children stated they couldn’t remember anything else about the event, but there was still information that was missing (i.e., some of the activities children experienced during the event were not mentioned). Here, the interviewer probed the child for the information that was missing, asking a series of focused questions. The number and content of these questions were dependent on the activities that the child had failed to remember during the open-ended recall phase. These were paired and followed-up with open prompts to encourage children to elaborate in their responses. For example, if in response to the focused question “Did you see a book that time?”, a child responded “Oh yeah. A book with flying frogs”, the interviewer would then ask “Tell me more about the book with flying frogs”. Before ending the interview by discussing a neutral topic, the interviewer once again asked whether the child remembered anything else about the event and after that, they were thanked for their efforts and participation.

A variety of supportive non-suggestive comments were used throughout the interviews. Interviewers expressed interest in the reported experiences (e.g., “I really want to know more about [reported experience”]), provided positive non-suggestive reinforcement (e.g., “You are really helping me understand what happened that day”), encouraged elaboration (e.g., “It is really important that you tell me everything you remember”) and offered reassurance (e.g., “Don’t worry. It’s ok that you don’t remember”). Interviewers also showed appreciation for the children’s efforts (e.g., “Thank you for telling me about that”) and used neutral facilitative comments (e.g., “ok”, “yes”, “uhuh”, “go on”, or repetition of the child’s last words). More details about the coding and categorisation of these comments are provided below.

## Data Coding

All interviews were video-recorded and transcribed verbatim. Coding focused on information that pertained to the target event (i.e., the substantive portion of the interview), therefore excluding any introductory exchanges at the beginning of the interview, attempts to establish rapport with the child, and attempts at the end of the interview to discuss neutral topics.

### Interviewer Prompts

Interviewer utterances were coded using the NICHD Interview Coding Scheme (Lamb et al. 2008) as invitations, cued invitations, directive, or option-posing and the total number of each type of utterances was recorded for each child. Focused/contaminating questions were also identified and totalled for each child. Each question type is described below.

*Invitations* referred to open-ended utterances using questions, statements, imperatives, or contextual cues to elicit narrative free-recall responses. These did not restrict the child’s focus except in a general sense. Invitations could also follow-up on information just mentioned or request additional free-recall elaboration about details previously mentioned (e.g., *Tell me everything that happened from the beginning to the end; Tell me more about that; Then what happened?*).

*Cued invitations* were utterances that refocused the child’s attention on previously mentioned details and used them as contextual cues in open-ended invitations to elicit narrative free-recall responses. Refocusing could be related to content cues (e.g., activities, objects, people, actions) mentioned by the child (e.g., *You mentioned [content mentioned by the child], tell me about that; Tell me everything that happened from [an occurrence*/*action mentioned by the child] until [another occurrence*/*action mentioned by the child]*).

*Directive* questions referred to utterances that focused on event-related information mentioned by the child earlier in the interview and requested additional information (or clarification) using a category, mostly wh- questions (who, what, when, where, how). Directive questions were “cued-recall” prompts (e.g., *Where*/*when did it happen? What colour was the puzzle?*).

*Option-posing prompts* were closed-ended questions that focused the child’s attention more narrowly on aspects of the event. They tapped recognition memory processes and could be formulated as yes/no or forced-choice questions (e.g., *Were the toys on the table when this happened? Were the toys inside the bag or on the table?*).

*Focused*/*contaminating* questions introduced event-related information (i.e., activities, aspects) that had not been previously disclosed by the child but did not imply that a particular response was expected (as suggestive questions would do). All focused/contaminating questions in this study asked about events or details that had occurred (as opposed to misleading questions) and varied depending on whether they were closed, requiring a yes or no answer (e.g., *Did you see a book that time?*), or whether they were open, requiring the children to provide the response (e.g., *I heard there was a book that time*.).

*Summary* statements focused children on what had already been mentioned and provide them with opportunities to elaborate and/or correct any information that had been misunderstood (e.g., *Let’s see if I understood everything that you told me*. (Pause). *You mentioned you did a puzzle*. (Pause). *And you played with some toys*. (Pause)…).

### Interviewer Supportiveness

Expressions of support in the substantive portion of the interviews were coded using an adaptation of the scheme developed by Hershkowitz et al. (2006). The total number of each type of utterance (supportive versus non-supportive prompts) was recorded for each child. The interviewer prompts were either supportive or neutral; thus, non-supportive prompts refer to the absence of supportive comments within interviewer utterances.

Expressions of *social support* referred to comments intended to unconditionally encourage children to be informative. These included addressing the child in a personal way, by using his/her name (e.g., *John, tell me everything about the book*); providing supportive non-suggestive positive reinforcement of the child’s behaviour during the interview that was unrelated to the content of their reports or to any other substantive issue (e.g., *You are remembering a lot*); providing comments showing appreciation for the child’s efforts and collaboration during the interview, but not specific contents (e.g., *Thank you for telling me about that*); and providing comments offering general reassurance (e.g., *That’s ok; Don’t worry*).

### Children’s Responses

In the current study, children’s recall of the personally experienced live event was assessed by counting the number of unique narrative details provided and by assessing their accuracy (i.e., the number of correct narrative details divided by the number of correct plus incorrect details).

Each unique narrative detail provided by the child was counted. A detail consisted of relevant words naming, identifying, or describing individual(s), object(s), event(s), place(s), and action(s) that were part of the event, as well as any of their features (e.g., appearance, location, time, duration, sound). Details expressing personal knowledge or habits (e.g., *I always wash my teeth like this*) were not counted. Each narrative detail provided was counted, but only when it was new and added to the understanding of the target event. For example, the following child response contained 38 new narrative details: “Yeah. There was a man fishing and he caught a fish and put it in his bucket. Then the cat took the fish out of the bucket and sort of walked away with it. And then there was a pelican, which took the fish out of the cat’s hand and put it in his mouth and flew away with it”.

Narrative details were counted for each question type separately and the total number of unique narrative details was tabulated, including details provided in response to all types of questions, i.e., the sum of the narrative details elicited by invitation, cued invitation, directive, option-posing and focused questions. When analysing children’s responses to supportive and non-supportive prompts the question types were collapsed and narrative details were counted for each prompt type separately (i.e., supportive prompts, non-supportive prompts) as was the total number of unique narrative details, including details provided in response to both types of prompts, i.e., the sum of the narrative details elicited by supportive and non-supportive prompts. Both the supportive and non-supportive prompts could be associated with any question type (i.e., invitations, cued invitations, directive and option-posing questions). Focused questions were not associated with supportive comments and thus this type of question was not included in any of the analyses regarding support.

We followed a child-oriented interview protocol and children chose the content of the information they wanted to provide. The number of questions of each type asked during the interviews was entirely dependent on the information previously disclosed by the child and, as a result, each child was asked a different number of each type of question. To take this into account in the analyses, the dependent variables were calculated by dividing the cell count of interest (e.g., total number of narrative details elicited using invitations in the interview) by the appropriate grouping total (e.g., total number of invitations asked in the interview), for each child. This gave us an average number of narrative details provided per prompt of each type (i.e., when an invitation was asked, children provided an average of X narrative details per prompt). This allowed us to explore whether different types of interviewer prompts were likely to elicit more information about the experienced event, regardless of how many prompts of each type were posed during the interview.

Similarly, supportive comments were included in some interviewer utterances (but not others) throughout the interview; as a result, each child received a different number of supportive or non-supportive questions. To take this into account in the analyses, our dependent variables regarding support were also calculated by dividing the cell count of interest (e.g., the total number of narrative details elicited by supportive prompts) by the appropriate grouping total (e.g., the total number of supportive prompts), for each child. This gave us an average number of narrative details provided per question of each type (i.e., when a supportive invitation was asked children provided an average of X narrative details, and so forth).

To determine the accuracy of the information supplied, we searched the video recordings of the event. Narrative details were coded as either correct or incorrect (i.e., errors of commission, such as describing a ball as red instead of blue, as well as reporting a piece of information that was not present or did not occur within the event). Details that could not be verified using the video recordings of the event were not scored. Percentage accuracy was determined by dividing the total number of correct narrative details recalled by the total number of narrative details recalled (i.e., correct + incorrect narrative details). As with the number of narrative details, the percentage accuracy was calculated for each question type and for each child.

## Reliability of Scoring

An independent rater scored 24 randomly selected interview transcripts (20% of the total). He was blind to the children’s diagnoses as well as the aims and hypotheses of the research but familiar with the template method of scoring used here. Cohen’s Kappa coefficients for agreement between raters for interviewer prompt types was K = 0.98, as it was for interviewer supportiveness (K = 0.98). Agreement was also high when identifying unique narrative details (K = 0.95) and verifying the accuracy of the narrative details provided by children (correct details K = 0.94; incorrect details K = 0.99). One of the raters, also blind to the child diagnosis, scored the remainder of the transcripts.

### Analysis Plan

First, we analysed of the effects of the interviewer prompts on the amount and accuracy of the information elicited. Research questions were addressed using a series of mixed-design analyses of variance (ANOVA), with Group entered as the between-subjects variable, and Prompt type and Delay entered as the within-subjects repeated-measures factors. For these analyses, we used the average number of narrative details recalled per question of each type. We then examined the effects of interviewer-provided support on the amount and accuracy of the information elicited. Research questions were addressed using a series of repeated measures ANOVAs, with Group entered as the between-subjects variable, and Support entered as the within-subjects factor. Separate analyses were carried out on the average numbers of correct narrative details, incorrect narrative details, and percentage accuracy. All parametric tests were conducted with child as the unit of analysis. All variables entered into parametric tests were normally distributed. Post hoc power analyses were conducted for each inferential test reported using G*Power version 3.1. When the assumption of sphericity was violated (Mauchly’s test), degrees of freedom were corrected using Greenhouse-Geisser estimates of sphericity. Effect sizes are indicated by partial eta-squared $\left( {\eta _{p}^{2}} \right)$. Simple effects analyses (with Bonferroni corrections) were used to unpack significant interactions. All statistical comparisons were two-tailed, using *p* < .05 as the level of significance.

## Results

### Preliminary Results

We analysed the substantive portions of the 118 interview transcripts. In total, an average of 49.47 (SD = 4.13, *n* = 5838) question-response pairs were identified in each transcript. Of these, an average of 36.00 (SD = 13.18, *n* = 4248, 72.76%) were substantive prompts, and 13.68 (SD = 9.49, *n* = 1587, 27.18%) were non-substantive prompts (i.e., procedural prompts or questions not related to the target event). Of the substantive prompts per interview, an average of 13.14 were invitations (SD = 3.95, *n* = 1551, 36.51%), 5.75 were cued invitations (SD = 2.48, *n* = 661, 15.56%), 1.26 were summary statements (SD = 0.93, *n* = 39, 0.92%), 6.70 were directive prompts (SD = 6.07, *n* = 771, 18.15%), 4.86 were option posing (SD = 3.98, *n* = 501, 11.79%) and 6.14 were focused/contaminating questions (SD = 3.12, *n* = 725, 17.07%).

The total number of prompts (invitations, cued invitations, directive, option-posing and focused/contaminating questions) given was totalled for each child. One-way ANOVAs with Group as the fixed factor were carried out for each time point, using the total number of prompts as the dependent variable. There was a significant main effect for Group in the two-week interview, *F*(1, 57) = 14.39, *p* < .001, and in the two-month interview, *F*(1, 57) = 10.53, *p* = .002. Two weeks after the experienced event, children with autism [*M* = 53.67, SD = 21.77, 95% CI (45.05, 62.28)] were given significantly more prompts than typically developing peers [*M* = 37.75, SD = 8.74, 95% CI (34.60, 40.90)]. Two months after the experienced event, children with autism [*M* = 45.85, SD = 13.89, 95% CI (40.36, 51.35)] were also given significantly more prompts than TD peers [*M* = 36.16, SD = 8.85, 95% CI (32.96, 39.35)].

Next, one-way ANOVAs with Group as a fixed factor was carried out for each time point using the total numbers of each type of prompt as the dependent variables. For directive questions, there was a significant main effect for Group in the two-week interview, *F*(1, 57) = 14.05, *p* < .001, and in the two-month interview, *F*(1, 57) = 12.39, *p* = .001. No significant main effects for Group were found for the remaining question types (invitations, cued invitations, option-posing, and focused questions) at both time points, all *F*s < 14.05, all *p*s > 0.077. In both interviews, children with autism [two weeks: *M* = 9.93, SD = 7.37, 95% CI (7.01, 12.84); two months: *M* = 8.85, SD = 7.51, 95% CI (5.88, 11.82)] were given significantly more directive prompts than TD peers [two weeks: *M* = 4.44, SD = 3.48, 95% CI (3.18, 5.69); two months: *M* = 3.81, SD = 2.81, 95% CI (2.80, 4.83)].

In total, 27% (n = 1289) of the interviewer prompts were supportive and 73% (n = 3535) were neutral/non-supportive. Autistic children received an average of 6.78 (SD = 3.56, range 1–17) supportive prompts per interview and typically developing children (TDC) received an average of 8.06 (SD = 3.05, range 2–15) supportive prompts per interview.

Two 5 (Prompt: invitations, cued invitations, directive, option-posing) × 2 (Group: ASD, TDC) repeated measures ANOVAs were carried out on the number of supportive prompts for each delay. Mauchly’s test indicated that the assumption of sphericity had been violated for the main effect of Prompt in the two-month interview, χ^2^ (5) = 12.65, *p* < .028, so degrees of freedom were corrected using Greenhouse–Geisser estimates of sphericity (ε = 0.66). There was a significant main effect of Prompt in the two-week interview, *F*(3, 45) = 4.70, *p* = .006, $\eta _{p}^{2}$ = 0.24, but not in the two-month interview, *F*(3, 27) = 1.16, *p* = .336, $\eta _{p}^{2}$ = 0.11. At both time points, there was no significant main effect of Group and no significant prompt × group interaction, all *F*s < 1.44, all *p*s > 0.245. In the two-week interview, children were given more supportive invitations [*M* = 3.79, SD = 2.30, 95% CI (2.60, 4.98)], than supportive directive prompts [*M* = 1.82, SD = 1.11, 95% CI (1.25, 2.39)]. No other significant differences were found.

Next, a 2 (Delay) × 2 (Group) repeated measures ANOVA was carried out on the number of supportive prompts. There was a significant main effect of Group, *F*(1, 56) = 5.02, *p* = .029, $\eta _{p}^{2}$ = 0.08, with TD children receiving more supportive prompts [*M* = 7.50, SD = 2.98, 95% CI (6.72, 8.28)] than children with ASD [*M* = 6.19, SD = 3.33, 95% CI (5.32, 7.06)] but there was no significant main effect of Delay *F*(1, 56) = 3.81, *p* = .056, $\eta _{p}^{2}$ = 0.064, and no significant Delay x Group interaction, *F*(1, 56) = 0.19, *p* = .668, $\eta _{p}^{2}$ = 0.003.

In sum, in both interviews, autistic children were given more prompts overall than TD peers. They were also given more directive prompts than TD children. Overall, TD children were given more supportive prompts than autistic children and in the two-week interview, children in both groups were given more supportive invitations than supportive directive prompts. Finally, discriminant function analyses revealed no significant effects for gender with respect to the number of narrative details remembered and the accuracy of those details and thus data were collapsed across gender for further analyses.

## Main Results

### Analyses of the Effects of Interviewer Prompts

Table 2 shows means and standard deviations for the average number of narrative details recalled per question asked (correct; incorrect) and the percentage accuracy of children’s recall, for each group and each prompt type (invitations, cued invitations, directive, option-posing, and focused questions).

**Table 2:** Recall informativeness and accuracy per question type

	Narrative details and group											
	Correct				Incorrect				% Accuracy			
	ASD		TDC		ASD	TDC			ASD		TDC	
Prompt type	*M*	SD	*M*	SD	*M*	SD	*M*	SD	*M*	SD	*M*	SD
Invitation	6.45	7.93	9.62	7.28*	0.78	1.80	1.62	1.65	0.88	0.11	0.87	0.11
Cued invitation	14.08	16.87	22.21	15.49*	1.94	5.48	3.84	5.04	0.88	0.17	0.86	0.15
Directive	5.14	10.07	9.61	9.25*	1.36	2.00	1.45	1.83	0.84	0.19	0.84	0.18
Option-posing	2.27	2.93	1.98	2.69	0.30	0.64	0.52	0.59	0.74	0.26	0.74	0.24
Focused/contaminating	5.34	3.36	4.60	3.08	1.30	1.21	1.02	1.11	0.83	0.19	0.84	0.18

Means and standard deviations for the average number of narrative details reported per question asked and percentage accuracy of children’s recall in response to each type of prompt by group and information type

ASD autism spectrum disorder, TDC typically developing children

*Significant group difference *p* < .05

### Correct narrative details

A 5 (Prompt) × 2 (Delay) × 2 (Group) mixed-design ANOVA was carried out on the number of correct narrative details recalled per question asked. Degrees of freedom were corrected using Greenhouse–Geisser estimates of sphericity for the main effect of Prompt, ε = 0.49 and the Prompt x Delay interaction, ε = 0.61. There was a significant main effect of Prompt, *F*(1.94, 110.49) = 68.21, *p* < .001, $\eta _{p}^{2}$ = 0.55, with cued invitations [*M* = 18.64, SD = 11.45, 95% CI (15.66, 21.63)] eliciting significantly more correct narrative details per prompt than any other prompt type. Invitations [*M* = 8.18, SD = 5.38, 95% CI (6.78, 9.59)] and directive prompts [*M* = 7.18, SD = 6.84, 95% CI (5.40, 8.97)] did not differ from each other and elicited more correct narrative details per prompt than option-posing questions [*M* = 1.99, SD = 1.99, 95% CI (1.47, 2.50)]. Focused/contaminating questions [*M* = 5.07, SD = 2.28, 95% CI (4.48, 5.67)] did not differ from directive questions and elicited significantly more correct narrative details per prompt than option-posing questions, but significantly fewer than invitations and cued invitations.

There was a significant main effect of Delay, *F*(1, 57) = 8.02, *p* < .006, $\eta _{p}^{2}$ = 0.12. Children provided significantly more information per question asked two weeks [*M* = 8.89, SD = 4.52, 95% CI (7.71, 10.07)] than two months [*M* = 7.54, SD = 4.21, 95% CI (6.44, 8.64)] after the event. There was also a significant main effect of Group, *F*(1, 57) = 7.35, *p* = .009, $\eta _{p}^{2}$ = 0.11, and a significant Prompt × Group interaction, *F*(4, 228) = 5.46, *p* < .001, $\eta _{p}^{2}$ = 0.09. Simple effects analyses examining the effects of Group on each prompt type revealed a significant Group effect on invitations, *F*(1, 57) = 6.24, *p* = .015, $\eta _{p}^{2}$ = 0.10, cued invitations *F*(1, 57) = 7.09, *p* = .010, $\eta _{p}^{2}$ = 0.11, and directive questions, *F*(1, 57) = 4.26, *p* = .044, $\eta _{p}^{2}$ = 0.07. Pairwise comparisons (*p* < .05, with a Bonferroni correction) showed that children with ASD reported significantly fewer correct narrative details per question asked in response to invitations, cued invitations and directive questions than did age-matched TD peers. Post hoc power analyses were conducted, which indicated that the mixed-design ANOVA had adequate power for all main effects and interactions (> 0.80).

### Incorrect Narrative Details

A 5 (Prompt) × 2 (Delay) × 2 (Group) mixed-design ANOVA was carried out on the number of incorrect narrative details recalled per question asked. Degrees of freedom were corrected using Greenhouse–Geisser estimates of sphericity for the main effect of Prompt, ε = 0.37 and the Prompt x Delay interaction, ε = 0.34. There was a significant main effect of Prompt, *F*(1.48, 84.43) = 7.30, *p* < .003, $\eta _{p}^{2}$ = 0.11, but no significant main effects of Group or Delay and no interactions, all *F*s < 1.75, all *p*s > 0.191. Children reported significantly fewer incorrect narrative details per question asked when prompted using option-posing questions [*M* = 0.41, SD = 0.44, 95% CI (0.24, 0.58)] than when prompted using invitations [*M* = 1.20, SD = 1.81, 95% CI (0.73, 1.67)], directive prompts [*M* = 1.41, SD = 2.00, 95% CI (0.88, 1.93)], or focused/contaminating questions [*M* = 1.16 SD = 0.82, 95% CI (0.84, 1.47)].

Post hoc power analyses indicated that the mixed-design ANOVA had adequate power for the main effects of Prompt, Group and interaction (> 0.80), but not for the main effect of Delay and interactions (< 0.80). Therefore, the effects of Delay on responses to each type of question was analysed further using a series of repeated measures ANOVAs, with Group entered as the between-subjects variable, and Delay entered as the within-subjects repeated-measures factor. The analyses revealed a significant main effect of Delay, *F*(1, 57) = 5.19, *p* = .027, $\eta _{p}^{2}$ = 0.08 for the amount of incorrect narrative details recalled per option-posing question asked, but not for the remaining types of questions. Children provided significantly more incorrect narrative details per option-posing question asked two months [*M* = 0.22 SD = 0.36, 95% CI (0.13, 0.31)] after the experienced event than they did after two weeks [*M* = 0.60 SD = 1.23, 95% CI (0.28, 0.92)]. No significant Delay × Group interactions emerged, all *F*s < 5.177, all *p*s > 0.072.

### Percentage Accuracy

A 5 (Prompt) × 2 (Delay) × 2 (Group) mixed-design ANOVA was carried out on the accuracy (percentage) of children’s recall for each of the questions asked. Degrees of freedom were corrected using Greenhouse–Geisser estimates of sphericity for the main effect of Prompt, ε = 0.77, and the Prompt × Delay interaction, ε = 0.62. There was a significant main effect of Prompt, *F*(3.06, 174.61) = 7.41, *p* = .001, $\eta _{p}^{2}$ = 0.115, but no significant main effects of Group or Delay and no interactions^2^, all *F*s < 2.14, all *p*s > 0.149. The narrative details elicited using invitations [*M* = 0.88 SD = 0.08, 95% CI (0.85, 0.91)] and cued invitations [*M* = 0.87 SD = 0.10, 95% CI (0.83, 0.91)] were significantly more accurate than those elicited using option-posing questions [*M* = 0.74 SD = 0.16, 95% CI (0.68, 0.80)]. Post hoc power analyses indicated that the mixed-design ANOVA had adequate power for the main effects of Prompt, Delay, and interactions (> 0.80), but not for the main effect of Group and the Prompt × Group interaction (< 0.80). Therefore, the effects of Group on the accuracy of children’s responses to each question type was analysed further in a 5 (Prompt) × 2 (Group) repeated measures ANOVA. The analyses also revealed a significant main effect of Prompt, which was described above, but no significant main effect of Group and no interaction^3^.

### Analyses of the Effects of Support

The effects of Group on recall were already reported in the previous analyses, thus we only report the effects of Support (as a within-subjects variable). Table 3 shows the means and standard deviations for the average number of narrative details recalled per question asked (correct; incorrect) and the percentage accuracy of children’s recall, for children in each group (ASD; TDC) and each type of prompt (supportive prompts; non-supportive prompts).

**Table 3:** Recall informativeness and accuracy in response to support

Details and delay	Group and interviewer supportiveness							
	ASD				TDC			
	Support		No support		Support		No support	
	*M*	SD	*M*	SD	*M*	SD	*M*	SD
Two-week interview								
Correct	8.69	11.48	7.45	6.73	17.30	11.48	11.51	6.73
Incorrect	1.05	2.97	1.08	1.42	1.96	2.96	1.28	1.42
Accuracy %	0.87	0.17	0.89	0.12	0.90	0.17	0.89	0.12
Two-month interview								
Correct	7.65	9.06	6.62	7.12	11.74	9.06	10.89	7.12
Incorrect	1.30	8.89	0.97	2.56	3.27	8.89	2.40	2.56
Accuracy %	0.81	0.21	0.87	0.12	0.86	0.21	0.84	0.12

Means and standard deviations for the average number of narrative details reported per question asked (correct; incorrect) and accuracy of children’s recall, by group and interviewer supportiveness

*ASD* autism spectrum disorder, *TDC* typically developing children

### Correct narrative details

Two 2 (Support) × 2 (Group) repeated measures ANOVAs were carried out on the number of correct narrative details recalled per prompt, one for each time point. There was a significant main effect of Support, *F*(1, 57) = 7.02, *p* = .010, $\eta _{p}^{2}$ = 0.11 in the two-week interview, but not in the two-month interview, *F*(1, 57) = 0.77, *p* = .383, $\eta _{p}^{2}$ = 0.01. Supportive interviewer prompts [*M* = 13.00, SD = 11.52, 95% CI (9.99, 16.00)] elicited significantly more correct narrative details per prompt than non-supportive prompts [*M* = 9.48, SD = 6.76, 95% CI (7.72, 11.24)] when the children were interviewed after two weeks. There were no significant interactions at either time point, all *F*s < 2.94, all *p*s > 0.092.

### Incorrect Narrative Details

Two 2 (Support) × 2 (Group) repeated measures ANOVAs were carried out on the number of incorrect narrative details recalled per prompt, one for each time point. The analyses revealed no significant main effects of Support in the two-week interview, *F*(1, 57) = 0.85, *p* = .360, $\eta _{p}^{2}$ = 0.02, or the two-month interview, *F*(1, 57) = 0.39, *p* = .534, $\eta _{p}^{2}$ = 0.01. Also, no significant Support × Group interactions were found, all *F*s < 1.02, all *p*s > 0.316.

### Percentage Accuracy

Two 2 (Support) × 2 (Group) repeated measures ANOVAs were carried out on percentage accuracy of the information provided, one for each time point. The analyses revealed no significant main effects of Support in the two-week interview, *F*(1, 57) = 0.26, *p* = .609, $\eta _{p}^{2}$ = 0.01, or the two-month interview *F*(1, 57) = 0.75, *p* = .392, $\eta _{p}^{2}$ = 0.01, and no significant Support × Group interactions^4^, all *F*s < 2.31, all *p*s > 0.134.

## Discussion

Our findings add to previous research demonstrating the effectiveness of using open-ended child-led recall prompts to elicit accurate accounts from vulnerable interviewees (Brown and Lamb 2015; Brown et al. 2012, 2015, 2017; Lamb et al. 2003). We showed that such prompts were also beneficial when interviewing cognitively and verbally able autistic children about their experiences. The present findings indicated that recall prompts elicited more detailed and accurate responses from children than recognition prompts. In particular, cued invitations elicited more detailed accounts than all other types of prompt, followed by invitations and directive prompts, which elicited similar amounts of information. Option-posing and focused/contaminating questions were the least effective for eliciting information about the event from children in both groups. Option-posing questions elicited significantly more errors after a longer delay.

A primary goal of the present study was to examine how autistic children would respond to various types of interviewer prompts and how effectively such prompts elicited new and accurate event-relevant information. In line with best practice recommendations, the Revised NICHD Protocol encourages interviewers to make extensive use of broadly open-ended prompts. Prompts such as invitations require episodic retrieval and involve autonoetic consciousness, i.e., the ability to use self-involvement to re-experience a past event in its full spatio-temporal context and mentally travel back in subjective time (Tulving 1985). There is evidence that autonoetic consciousness (i.e., self-knowing) is reduced in ASD, due to a poorly developed level of self-awareness, which leads to difficulties in remembering personally experienced events (Lind and Bowler 2008). Thus, based on the empirical observations of impaired episodic memory (Boucher 1981; Lind and Bowler 2008; Loth et al. 2011; McCrory et al. 2007), difficulties in the free recall of information (Bennetto et al. 1996; Boucher and Warrington 1976; Bowler et al. 1997; Maras et al. 2012), and difficulties in the recall of personally experienced events (Crane and Goddard 2008; Hare et al. 2007; Klein et al. 1999; Lind and Bowler 2010; Maras and Bowler 2012, 2013) we expected that autistic children would find it more difficult than TD peers did to recall what happened in response to broadly open-ended prompts, such as “Tell me everything that happened”.

As anticipated, autistic children recalled fewer correct narrative details about the experienced event than TD children in response to input-free recall based-prompts (i.e., invitations and cued invitations). A possible explanation for their poorer free recall is that autistic children found it difficult to conduct the cognitively demanding task of mentally traveling back in time to search for information about the event, as required to access episodic memory (Tulving 2002), whilst also having to determine what aspects of the past event were relevant to the demands contained in open-ended free recall prompts such as “tell me *everything* that happened” (Loth et al. 2008). Deficits in executive functioning and working memory in autistic individuals are well established (e.g., Bennetto et al. 1996; Hill 2004) and can influence children’s ability to perform in cognitively demanding and complex tasks (Minshew and Goldstein 2001; Poirier et al. 2011; Williams et al. 2006), particularly when unsupported at retrieval (Bowler et al. 2008).

On the other hand, and contrary to our predictions, we found that invitations elicited just as many correct narrative details per prompt from children with ASD as open-ended directive prompts. Similarly, the information recalled by autistic children in response to open-ended invitations was just as accurate as that recalled in response to more specific (directive) questions. The questions coded as *directive* in this study refer to focused recall questions, based on previously disclosed details, that requested specific categories of information (e.g., “Where was the ball?”). These questions have been named differently in laboratory studies of individuals with ASD, typically as “cued recall” or “specific/guided” questions, and the information provided in response to such questions by ASD individuals has been associated with equivalent levels of accuracy as that provided by TD peers (Bowler et al. 2008; Maras and Bowler 2010; Maras et al. 2012, 2013; Millward et al. 2000). We too found that intellectually and linguistically able autistic children were indistinguishable from TD peers with respect to the accuracy of their recall in response to directive questions.

Most noticeably, our study demonstrated the particular effectiveness of *cued invitations*, which are a distinctive feature of the NICHD Protocol, when interviewing autistic children about their experiences. Cued invitations are follow-up open-ended prompts that refocus the child’s attention on previously mentioned details (in their own words) and use them as contextual cues to elicit narrative or multiword responses (“Earlier you mentioned that you read a book with flying frogs. Tell me everything about the book with flying frogs.”). The usefulness of this specific cuing strategy has been demonstrated in field and laboratory analogue studies with vulnerable children (Brown et al. 2013; Cederborg et al. 2008; Cederborg and Lamb 2008; Lamb et al. 2003) and our study provides further evidence of its effectiveness.

Although autistic children recalled fewer details than TD peers in response to cued invitations, the information elicited using such prompts comprised core and accurate details about their experiences, even when a substantial amount of time had elapsed between the event and the interview (91% accurate in the two-week interview and 86% in the two-month interview). Moreover, when prompted using cued invitations, autistic children recalled more (and more accurate) information than when prompted using any other type of prompt. It has been argued that more supportive retrieval techniques may aid witnesses with ASD to recall more information, possibly because their recall impairments are more related to retrieval than to encoding mechanisms (Maras and Bowler 2013). The current findings supported the notion that cued invitations constitute effective ways of triggering the recall of information and enhance the capacity of children with ASD to elaborate upon their narrative accounts, by structuring recall of experienced events, associating them with pre-disclosed actions, and breaking them into smaller units.

Our results offered further evidence that open-ended recall-based prompts, particularly cued invitations, promote complete and accurate eyewitness recall in cognitively and verbally able autistic children by fostering further elaboration of previously disclosed information. They also suggested that cognitively and verbally able autistic children were as capable as TD children, with respect to the elaboration and accuracy of their recall, when they were questioned in a supportive manner, and in accordance with best practice recommendations. Autistic children may require more prompts than TD children to elicit the same amount of information, but these prompts do not need to be, and should not be, more specific, restrictive, or closed. It is important to note at this point that communicating expectations clearly and motivating children to provide as much information as they could might have influenced their performance as well. As mentioned before, literal and concrete thinking is common in autistic individuals, so framing each question/statement as directly and clearly as possible and explaining the specific level of detail that was expected of them likely helped ensure that children understood the unique demands of the interview context (Sternberg et al. 2002). As research demonstrates, the complexity of the language and questions addressed to children can strongly dictate the course and outcome of investigative interviews (Lamb et al. 2015).

We found no evidence that autistic children were more likely to report erroneous information than TD peers. However, although children reported few incorrect narrative details overall, these were more frequently included in children’s responses to recall-based prompts (invitations, cued invitations and directive prompts). Thus, all those involved in the criminal justice system should be aware that the use of such prompts might elicit extremely valuable and uncontaminated information about an event but might also lead children to include a small amount of incorrect information in their detailed responses.

All types of prompts elicited forensically relevant and accurate information from children, and our findings indicated that accuracy remained high even when the interviewer used prompts associated with higher error rates, such as option-posing prompts (Home Office 2011; Lamb et al. 2008, 2018). This finding should, however, be interpreted with caution because recognition-based (forced-choice and yes/no) questions were the least productive prompts and were associated with a significant increase in the amount of erroneous information reported by children in both groups during the two-month interview. These findings are consistent with empirical evidence demonstrating that closed prompts (e.g., yes/no or multiple choice questions) elicit less detailed, coherent and organized responses, as well as more errors and inconsistent statements than any of the other types of prompts (Cederborg et al. 2000; Feltis et al. 2010; Korkman et al. 2006; Lamb and Fauchier 2001; Waterman et al. 2000). There is also evidence of deficits in recognition memory by autistic individuals, especially when forced-choice rather than yes/no testing procedures are used (Bowler et al. 2004).

Furthermore, our results showed that accuracy remained high when interviewers asked focused/contaminating questions. After a two-month delay, the accounts provided by children with ASD were no less accurate in response to focused/contaminating prompts than after a two-week delay and were no less accurate than those provided by TD children. This is consistent with other reports that, when asked focused questions, cognitively and verbally able children and adults with ASD were no more suggestible than their typical counterparts (e.g., Bruck et al. 2007; Maras and Bowler 2011; McCrory et al. 2007; North et al. 2008). However, our study did not directly assess suggestibility and the focused questions asked in the present study did not include questions as risky as suggestive confrontational (e.g., “Are you lying?”), or tag questions (e.g., “She told you to assemble a puzzle, didn’t she?”), which, if used, might have had a more damaging effect on accuracy.

Additionally, children in our study were not exposed to misinformation about the event and did not, according to their parents, discuss what happened during the event with other people. Individuals with ASD show impairments in executive function (Hill 2004) and language comprehension (e.g., Henderson et al. 2011; Jolliffe and Baron-Cohen 1999; Norbury 2005), which might make them more suggestible and vulnerable to contamination. Further research on this would thus be valuable.

For children in both groups, supportive interviewer prompts elicited significantly more correct narrative details than non-supportive prompts, but, contrary to our predictions, social support was particularly beneficial when children were interviewed for the first time and not after a longer delay. After a delay of two months, social support did not seem to affect children’s responses. Importantly, there was no evidence that social support adversely affected children’s accuracy either.

Empirical evidence generated in laboratory and field research has demonstrated that supportive interviewing techniques are associated with decreases in children’s anxiety and, as a result, increases in children’s responsiveness, cooperation, and the amount of relevant information provided (e.g., Hershkowitz 2006, 2009; Rush et al. 2014). We too found that, after a short delay, children were more informative when the interviewer prompts contained supportive comments, without a concomitant increase in errors or decrease in accuracy. The first interview corresponded to an unfamiliar situation outside children’s usual routine and the supportive behaviour of the interviewer (e.g., calling the child’s name, thanking him/her for the information provided), might have helped to mitigate possible feelings of anxiety and discomfort that could otherwise have adversely affected children’s performance during the interview.

However, whereas Bottoms et al. (2007) found that social support increased the amount of correct and decreased the amount of incorrect information reported by children one year after an experienced event, our results demonstrated that interviewer support did not have an effect on children’s responses after a two month delay. This is in keeping with Imhoff’s (2000) findings that interviewer support did not affect children’s responses after a four-week delay. The lack of effect of social support during the second interview may be partially explained by children’s previous exposure not only to the interviewer, but also to the interview setting, which progressively became a less strange, unfamiliar situation. Interviewers can build trust across repeated interviews and, as a result, lessen children’s anxiety (Pipe et al. 2007).

Our findings seem to indicate that children in the two groups might have been differentially affected by supportive interviewing, as there were substantial mean performance differences during the two-week interview (although these did not reach statistical significance). Thus, it remains unclear whether autistic children benefit from the same improvements to memory performance in response to social support as that reported by previous research for TD children. Our results did, however, clearly demonstrate that interviewer-provided social support did not adversely affect children’s accuracy and, soon after the event, increased the amount of correct information recalled. These findings have important practical implications for the legal system, amongst others, suggesting that, by employing a more supportive attitude during questioning, interviewers may help make the interview process more tolerable for children, without negatively affecting the quality of their testimony. Of course, whether or not interviewer-provided social support actually changes children’s memory performance can only be fully demonstrated in experiments that include supportive and non-supportive control conditions and thus, further research would be valuable.

As in most laboratory eyewitness research there are some limitations to acknowledge. Our clinical sample comprised cognitively and verbally able children with ASD, and we cannot be certain that the same tendencies would be observed among less intellectually capable children with ASD. It is extremely important that future research include individuals on the autism spectrum whose intellectual or verbal ability is below the normal range. Unfortunately, practical constraints prevented us from testing verbal or full-scale IQ, but only children with ASD and intellectual and linguistic abilities within the normal range (i.e., verbal quotients of 85 or above and full-scale IQ of 90 or above) were referred to us for participation, and the typically developing children did not have any symptomology or known psychiatric, developmental, or neurological disorders.

Our sample size was comparable to that in previously published studies involving individuals with ASD, but it is relatively small by the standards of psychology more generally. It is important to replicate our findings using a larger sample, to allow the full examination of more complex interactions between variables. It is also important to note that the constraints involved in conducting a staged event for an experimental study meant that the children were questioned about a neutral standardized event, so generalising these findings to stressful real events (such as abusive incidents) may not be warranted. However, although we used a neutral and standardized event, it is possible that the session was more distinctive and potentially emotionally arousing for children in the ASD group, as it was associated with the moment of their ASD diagnosis. Future research exploring memory for both positively and negatively arousing experienced events by children with ASD would be valuable.

Most of the interviews were carried out in a different room, and in some cases in a different building, from the event-to-be-recalled, thereby avoiding context reinstatement, but this was not always possible. Previous research has suggested that returning to the same physical environment where the target event took place can enhance recall in adults with ASD (Maras and Bowler 2012). Repeated interviews can also provide effective reinstatement for typically developing children because they foster systematic and detailed recall of a target event (La Rooy et al. 2005), so it would be valuable to explore this issue in future research involving children with ASD. Finally, the cued recall questioning phase did not focus on what the child freely recalled but on what the interviewer knew had occurred. In real life, of course, a careful interviewer can only base questions on what the witness has previously recalled.

Our research was a first step toward understanding the effect of interviewer supportiveness on the amount and accuracy of children with ASD’s accounts of personally experienced events after two-time delays. However, it did not include the necessary experimental conditions to fully test the effects of interviewer supportiveness. In future research, it would be important to include “intimidating”, “neutral” and “supportive” interview conditions to fully test the effects of social support. With the exception of four cases, the same interviewer conducted both interviews as well, and this may have diluted the effects of supportive behaviour in the second interview. Further research should also consider possible carry-over effects of interviewer support from previous questions. Finally, we used a modification of the Revised NICHD Protocol to accommodate the social communication deficits often displayed by children with autism, and it remains to be seen whether other supportive strategies would have similar effects.

Notwithstanding these limitations, the current study showed that, although cognitively and verbally able children with ASD may require more prompts than TD children to provide the same amount of information, they can provide meaningful and reliable accounts of their experiences, when appropriately questioned, even after lengthy delays. The findings reported above show that the best-practice principles embodied in the Revised NICHD Protocol, an open-ended child-oriented interview guide, effectively promote accurate remembering and responding by children on the autism spectrum. It is essential to continue investigating valid strategies and empirically validated retrieval support tools that can aid young witnesses with ASD to freely recall as much information as possible about past experiences when they come into contact with the criminal justice system. As previously highlighted, it is critical to explore ways to accommodate the sensory needs of children with autism in court, develop appropriate techniques to help them feel more comfortable and less anxious in legal settings, and encourage them to give elaborate and reliable accounts of past experiences.

## Footnotes

1 To respect the multiplicity of views regarding how individuals with autism should be described, in this article we use both identity-first language (i.e., autistic children) as well as person-first language (i.e., children with autism). Many professionals in the scientific and medical community endorse the use of person-first language (e.g. “person with autism”), while members of the autism community (autistic people, caregivers, and broader support networks) favour identity-first terms, such as “autistic” or “autistic person” (Gernsbacher 2017; Kenny et al. 2016).

2 The same results emerged when the percentage accuracy scores were subjected to arcsine transformations.

3 The same results emerged when the percentage accuracy scores were subjected to arcsine transformations.

4 The same results emerged when the percentage accuracy scores were subjected to arcsine transformations.
